# Identification of reproducible gene expression signatures in lung adenocarcinoma

**DOI:** 10.1186/1471-2105-14-371

**Published:** 2013-12-26

**Authors:** Tzu-Pin Lu, Eric Y Chuang, James J Chen

**Affiliations:** 1 Division of Bioinformatics and Biostatistics, National Center for Toxicological Research, Food and Drug Administration Jefferson, Little Rock, Arkansas, USA; 2 YongLin Biomedical Engineering Center, National Taiwan University, Taipei, Taiwan; 3 Graduate Institute of Biomedical Engineering and Bioinformatics National Taiwan University, Taipei, Taiwan; 4 Graduate Institute of Biostatistics and Biostatistics Center, China Medical University, Taichung, Taiwan

**Keywords:** Lung adenocarcinoma, Microarray, Pathway analysis, Prognostic biomarker, Predictive biomarker

## Abstract

**Background:**

Lung cancer is the leading cause of cancer-related death worldwide. Tremendous research efforts have been devoted to improving treatment procedures, but the average five-year overall survival rates are still less than 20%. Many biomarkers have been identified for predicting survival; challenges arise, however, in translating the findings into clinical practice due to their inconsistency and irreproducibility. In this study, we proposed an approach by identifying predictive genes through pathways.

**Results:**

The microarrays from Shedden et al. were used as the training set, and the log-rank test was performed to select potential signature genes. We focused on 24 cancer-related pathways from 4 biological databases. A scoring scheme was developed by the Cox hazard regression model, and patients were divided into two groups based on the medians. Subsequently, their predictability and generalizability were evaluated by the 2-fold cross-validation and a resampling test in 4 independent datasets, respectively. A set of 16 genes related to apoptosis execution was demonstrated to have good predictability as well as generalizability in more than 700 lung adenocarcinoma patients and was reproducible in 4 independent datasets. This signature set was shown to have superior performances compared to 6 other published signatures. Furthermore, the corresponding risk scores derived from the set were found to associate with the efficacy of the anti-cancer drug ZD-6474 targeting *EGFR*.

**Conclusions:**

In summary, we presented a new approach to identify reproducible survival predictors for lung adenocarcinoma, and the identified genes may serve as both prognostic and predictive biomarkers in the future.

## Background

Lung cancer is the leading cause of cancer-related death in the United States and worldwide [1,2], more than 25% of cancer-related deaths in both men and women resulted from lung cancer. In the past few decades, considerable research effort has been devoted to elucidating the etiology of lung cancer and identifying possible biomarkers for predicting survival outcomes [3-6]. However, the overall five-year survival rate for lung cancer is still less than 20%. Although several studies have identified expression signatures associated with survival outcomes in their original discovery datasets, these expression signatures were often found to be irreproducible across studies. A previous report has shown that the largest number of overlapping predictive genes between two independent studies was only four, and most often even zero [7]. The lack of reproducibility in independent studies makes it very difficult to justify conducting prospective clinical validation studies and application to clinical practice. Therefore, how to effectively identify a set of predictive and reproducible biomarkers across different lung cancer studies poses a major challenge.

To address this issue, one possible approach for biomarker identification is to perform analysis based on biologically functional relevant gene sets rather than to examine the associations by traditional gene-by-gene approaches. Several reports have demonstrated that predictive genes that were identified by approaches considering biological pathway or cellular function information are more often reproduced and validated in independent studies [8-11]. For example, Kang et al. selected 23 effective genes to predict patient survival outcomes and chemotherapy responses in ovarian cancer from a pool of 151 genes summarized by DNA repair pathways [8]. An approach based on functional gene sets not only provides better understanding of biological meaning of identified gene markers, but also greatly increases statistical power of identification by considering only the number of pathways rather than the number of genes in the multiple comparison adjustment. The approach to performing gene set enrichment analyses for gene expression profiling has become a popular methodology even for genome-wide association studies [12,13].

In a recent study of an analysis of 47 published gene expression signatures for breast cancer [14], the authors showed that the predictive performance of several signatures was not superior to that obtained from random gene expression signatures, and sometimes even worse. There was a tendency for significant marker sets to depend on the size of the set; and over 90% of random signatures with more than 100 gene members were significant predictors for survival outcome [14]. This might be attributed to a lack of independence among the genes in the array, and a predictive model tending to become significant as more gene members were included. In order to reduce the possibility of identifying false-positive signatures, resample techniques can be performed to demonstrate significant findings, as compared to a randomly selected signature set with a same number of genes.

Genomic signatures can be classified into two major types: prognostic biomarkers and predictive biomarkers. Prognostic biomarkers aim to help physicians divide patients into distinct groups based on their risk factors and disease characteristics, which hints that different drug treatments including standard treatment, or advanced aggressive therapeutic procedures may be applied to groups of patients showing poor survival outcomes. Predictive biomarkers for a specific treatment are able to identify a subgroup of suitable patients for receiving the treatment, and those patients expected to have poor responses would be spared from the treatment. Compared with prognostic biomarkers, predictive biomarkers provide physicians direct suggestions for specific therapeutic methods for individual patients. Currently, most identified (published) signatures in lung cancer fall into the category of prognostic biomarkers; the lack of studies for predictive biomarkers is partly due to many considerations required before conducting a treatment-specific randomized clinical trial. Recently, Barretina et al. [15] used microarrays to investigate the gene expression profiles of 479 human cancer cell lines along with their responses after exposure to 24 anticancer drugs. Among the 24 drugs, 3 were designed to directly target *EGFR*, whose association with lung cancer has been widely reported [16-18]. This dataset provides a good template for evaluation of predictive biomarkers in lung cancer.

In the development of a predictive signature, there are two primary assessment considerations: 1) predictability (predictive performance) and 2) generalizability (reproducibility). Predictability is the ability of the signature to predict survival risk scores of patients who were not included in the signature development, but who are from a similar population. To evaluate predictability is to determine whether the signature can provide a relative ranking of patient risk scores correctly. A predictive signature that performed well in the development study might not be reproduced in performance when applied to other studies. Generalizability is to determine whether predictability is reproducible when applied to data generated independently in different circumstances, such as different locations or different times. A predictive signature needs to establish its predictability and generalizability before a prospective clinical validation is conducted.

In this study, we propose an approach for identifying prognostic gene signatures using 442 lung adenocarcinoma patients from the study of Shedden et al. [5]. The approach consists of a two-stage assessment: predictability and generalizability. The Shedden dataset was used for predictability assessment, and 4 additional independent lung cancer datasets were used for generalizability assessment. The Shedden (training) dataset was tested using 2-fold cross-validation. Generalizability of signature sets was evaluated and compared with several published lung cancer prognostic signatures using a randomization test. A set of 16 genes involved in the function of apoptotic execution phase was shown to have good predictability as well as generalizability in more than 700 lung adenocarcinoma patients, including the 442 patients from the Shedden et al. study. This signature set was also found to associate with the efficacy of the anti-cancer drug ZD-6474 targeting *EGFR*.

## Results

### Identification of significant survival predictors

Among the 12,753 genes investigated in the dataset of Shedden et al., log-rank tests were performed to exclude those genes determined to have no association with survival (*p* > 0.1, Step 1). A total of 2,852 genes (22.36%) were selected and their functions were annotated by four biological databases, including KEGG, BioCarta, PID and Reactome. We focused on 24 pathways that were closely related to the development of cancer cells, involving apoptosis, cell cycle and cell death regulations (Additional file 1: Table S1, Step 2).

For each pathway, the risk score of each patient was computed, and patients were divided into two groups according to the procedure described in the Method section. The log-rank test was performed to determine statistical significance of the signature set associated with survival outcomes for the pathway. Table 1 shows the p-values of 16 pathways that were significant using the Bonferroni correction (α = 0.05/24). A randomization test was performed and all 16 pathways were highly significant (results not shown).

**Table 1:** Predictive performances of 16 pathways

**Pathway**	**Database**	**Number of genes**^**a**^	***p***-**value**^**b**^	**Count**^**c**^	**Count**^**d**^
Apoptosis reactome	Reactome	43	7.58E-06	36	50
Polo-like kinase signaling events in the cell cycle	PID	42	5.86E-05	34	73
Apoptotic execution phase	Reactome	16	1.16E-05	27	48
Intrinsic pathway for apoptosis	Reactome	9	2.22E-06	26	47
Apoptosis KEGG	KEGG	16	6.50E-06	24	31
Biocarta cell cycle pathway	BioCarta	7	2.53E-08	23	47
Cell cycle	KEGG	46	1.64E-04	21	67
APC C-mediated degradation of cell cycle proteins	Reactome	32	3.27E-05	16	57
Regulation of mitotic cell cycle	Reactome	32	3.27E-05	16	57
Cell cycle mitotic	Reactome	60	3.63E-04	12	72
Caspase cascade in apoptosis	PID	9	4.82E-04	12	32
NRAGE signals death through JNK	Reactome	7	1.32E-05	7	25
Cell death signalling via NRAGE NRIF and NADE	Reactome	8	1.53E-04	4	15
Biocarta death pathway	BioCarta	9	5.82E-04	4	19
Apoptotic cleavage of cellular proteins	Reactome	12	4.06E-05	3	17
Regulation of apoptosis	Reactome	18	2.01E-05	1	9

^a^Only genes showing association with survival (*P* < 0.1) were analyzed.

^b^Estimated by using log-rank tests.

^c^The frequency showing *p*-values < 0.002083 in the 100 trials of the internal validation assessment.

^d^The frequency showing *p*-values < 0.05 in the 100 trials of the internal validation assessment.

### Predictability assessment (analytic validation of the training set)

Internal analytical validation was performed on the Shedden study to assess predictability of the signature sets using 2-fold cross-validation. The 442 samples were randomly divided into two (sub) sets of equal size, a training and a test set. In the training phase, gene filtering, Cox model fitting, total risk score calculation, and log-rank test were performed according to the procedures described in the Method section. The median of the risk scores was computed for each pathway.

In the test phase, a score of 0 was initially assigned to each patient. For each gene, a score of 1 was given to a patient if the Cox coefficient from the training set was greater than 0 and the patient’s expression level was higher or equal to the median, or if the coefficient was less than 0 and the expression level was less than the median. The total risk score of each patient was computed for each pathway (Step 3). Next, patients were classified into two risk groups based on the median of the risk scores from the training set. The log-rank test was performed to compare the two risk groups at the significance level of 0.05 (Step 4). Here we considered both the unadjusted p-value (α = 0.05) and the Bonferroni adjusted p-value (*α* = 0.05/24). The procedure was repeated, but the model was developed on the test set and applied to the training set. The entire procedure was repeated 50 times to generate different 2-fold partitions. The frequency of significant results out of the 100 repetitions was calculated for each pathway. The last two columns of Table 1 list the frequencies of the significant results for the 16 pathways. We selected the top 11 pathways which showed at least 10 significant counts at *α* = 0.05/24 (and at least 30 significant count at *α* = 0.05) for the generalizability assessment. These numbers can be regarded as the power for the predictability.

### Generalizability assessment (analytic validation of the external datasets)

Generalizability requires that predictive performance of a model developed from one study is reproducible across different studies. Five additional microarray studies for a total of 6 studies (Additional file 1: Table S2) were evaluated. Among the 6 studies considered, 5 studies contained adenocarcinoma samples and 4 studies contained squamous cell carcinoma samples (i.e., 3 studies contained both adenocarcinoma and squamous data). Evaluation of generalizability is similar to the evaluation of predictability in which all 442 samples from the Shedden study were used as the training dataset and applied to each of 4 independent studies as a test dataset. Briefly, four microarray datasets were retrieved from the Gene Expression Omnibus (GEO) [19], and the quantile normalization algorithm was utilized to remove systematic biases. Gene expression levels of multiple probes annotated with the same gene symbol were summarized into a single value by using their means. Each independent dataset was then evaluated using the procedures described in the assessment of predictability.

Table 2 shows the p-values and the number of significant results (p ≤ 0.05) for the 11 most significant pathways (p ≤ 0.05/24) for the five adenocarcinoma datasets, including Shedden and four independent datasets. Several pathways were significant for 3 or more datasets; the pathway “apoptotic execution phase” was significant for all 5 datasets, an excellent criterion for generalizability.

**Table 2:** **Predictive performances estimated by the log**-**rank tests of the 11 pathways for five lung adenocarcinoma datasets**

**Pathway (Gene Number)**	**Database**	**Shedden**	**GSE3141**^**a**^	**GSE8894**^**a**^	**GSE11969**^**a**^	**Beer**	**Significance**	**Resampling**
Apoptotic execution phase (16)	Reactome	1.16E-05	0.0038	0.0002	0.0008	0.0332	5	0.000
Biocarta cell cycle pathway (7)	Biocarta	2.53E-08	0.0596	0.0012	0.025	0.1527	3	0.006
Cell cycle (46)	KEGG	1.64E-04	0.0214	0.0050	0.0057	0.2008	4	0.053
Apoptosis (16)	KEGG	6.50E-06	0.673	0.0191	0.0838	0.0468	3	0.059
Caspase cascade in apoptosis (9)	PID	4.82E-04	0.2174	0.2737	0.3716	0.0055	2	0.077
APC C-mediated degradation of cell cycle proteins (32)	Reactome	3.27E-05	0.2322	0.0120	0.0075	0.5752	3	0.159
Regulation of mitotic cell cycle (32)	Reactome	3.27E-05	0.2322	0.0120	0.0075	0.5752	3	0.159
Polo-like kinase signaling events in the cell cycle (42)	PID	5.86E-05	0.4168	0.0298	0.0389	0.229	3	0.192
Intrinsic pathway for apoptosis (9)	Reactome	2.22E-06	0.1404	0.3642	0.9689	0.4496	1	0.198
Apoptosis (43)	Reactome	7.58E-06	0.0136	0.0598	0.0112	0.3525	3	0.211
Cell cycle mitotic (60)	Reactome	1.16E-05	0.2992	0.0008	0.0007	0.085	3	0.281

^a^Only adenocarcinoma patients were analyzed here.

In addition, a resampling test was performed to simulate chance significance for the five adenocarcinoma datasets. For each pathway, a null signature set consisting of randomly selected genes from the Shedden study was generated, where the numbers of genes were kept the same as the pathway. The null signature set was applied to the five datasets; the total number of significant results was compared to the observed number of significant results in Column 8 to determine if the selected signature set was superior to the null signature set. The simulation was repeated 1,000 times. The last column shows the p-value for the 11 pathways, where the p-value was defined as the proportion of the number of significances from the null signature set was greater or equal to the observed number in Column 8 out of the 1,000 repetitions.

The 11 pathways were also applied to four squamous cell carcinoma datasets, GSE3141, GSE4573, GSE8894, and GSE11969, and no statistical significance was identified for any of 11 pathways and 4 datasets (Additional file 1: Table S3). This suggested that these 11 pathways were specific to the lung adenocarcinoma.

### The 16-gene signature

**Figure 1:**
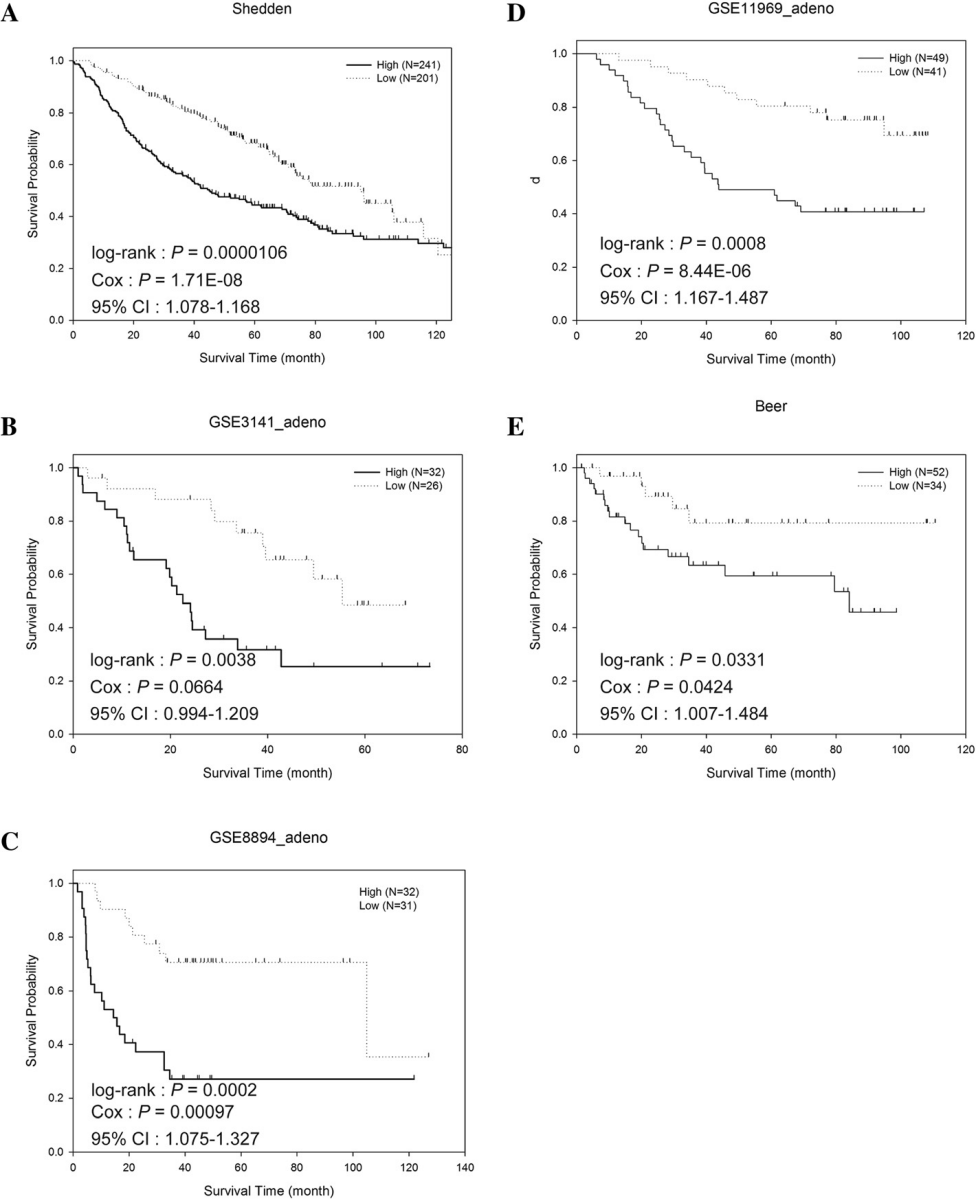
**Kaplan**-**Meier survival curves of lung adenocarcinoma patients divided by their summarized scores.** For each dataset, the patients were classified into “High” or “Low” groups based on their median score. **(a)** Shedden et al. **(b)** GSE3141 **(c)** GSE8894 **(d)** GSE11969 **(e)** Beer et al.

The 16 genes involved in the apoptotic execution phase pathway and statistical *p*-values of the log-rank test from Step 1 are listed in Table 3. Among them, the most significant gene was *DNM1L*, which concurred with high expression of *PKP3* and *DNM1L* associated with poor survival in lung adenocarcinoma patients [20]. Figure 1 shows the Kaplan–Meier survival curves with the p-values from the log-rank test for the datasets. The proposed calculation of risk scores scheme and classification of patients into “High” and “Low” groups appeared to perform well in both training and test data. For example, in the study of Shedden et al., the median survival of the patients belonging to the “High” group is obviously shorter than that from the “Low” group (44.52 month versus 94.35 month). Similarly, the median survival also showed dramatic differences between the “High” and “Low” groups in GSE8894 (17.55 month versus 117.41 month). The p-values from fitting the Cox hazard regression model using the risk scores as an independent variable are shown in Figure 1. The p-values were consistent with the p-values from the log-rank test; a minor discrepancy is that the dataset GSE3141 has 6 ties at the median score resulting in lower discriminating ability. We also performed an analysis dividing the patients into three groups (Additional file 1: Figure S1), and an analysis of the squamous cell carcinoma patients across 4 independent datasets. The results are consistent with the results present above.

**Table 3:** **List of the 16 genes identified and their p**-**values from the log**-**rank test**

**Gene**	**Association**^**a**^	***p***-**value**	**Gene**	**Association**^**a**^	***p***-**value**
*ADD1*	-	2.26E-02	*OCLN*	-	1.72E-02
*DNM1L*	+	4.70E-04	*PAK2*	+	4.56E-02
*DSG2*	+	1.63E-03	*PKP1*	+	5.65E-02
*DSP*	+	1.24E-02	*PRKCD*	-	4.95E-03
*HMGB2*	+	3.97E-03	*PRKCQ*	-	3.18E-02
*KPNB1*	+	4.25E-02	*SATB1*	-	7.72E-02
*LMNB1*	+	4.85E-02	*STK24*	+	1.87E-02
*MAPT*	-	9.91E-03	*TJP1*	+	7.72E-02

^a^“+” denotes the fitted Cox efficient > 0 whereas “-“denotes the fitted Cox coefficient < 0.

The risk scores and four clinical variables were analyzed simultaneously using the multivariate Cox regression. The four clinical variables were age, gender, smoking, and grade (Additional file 1: Table S4). Additional file 1: Table S5 shows the coefficients of the fitted Cox regression. The proposed risk score was the most significant predictor as compared with the other four clinical variables.

### Comparisons of the 16-gene signature with published lung cancer prognostic signatures

The predictive performances of the 16 gene signature were also compared with the published lung cancer prognostic signatures. Six signature sets from five studies were considered [3-5,21-23]. We collected the gene symbols from their original literatures and followed the same evaluation procedures described previously to test their predictability. The results are summarized in Table 4; it appeared that the 16-gene signature identified in this study was superior to those identified in other studies. Specifically, among the published signatures, the best performance was observed in the 10 genes reported in the study of Kratz et al. [24]. These 10 genes were able to predict four datasets with one on the border line (*P* = 0.12). The other signatures were effective predictors for 2 or 3 datasets. Therefore, the results suggested that our analysis approach not only was able to reproduce published gene signatures from other studies but also identified a set of predictive genes for lung adenocarcinoma.

**Table 4:** **Comparisons of the predictive performances of the 16**-**gene signature with 6 published lung cancer signatures**

**Signature**	**Number**	**Ref**	**Shedden**	**GSE3141**^**a**^	**GSE8894**^**a**^	**GSE11969**^**a**^	**Beer**
This Study	16	--	1.16E-05	0.0038	0.0002	0.0008	0.0332
Chen et al.	5	[25]	0.0244	0.7504	0.0050	0.0954	0.9578
Chen et al.	15^b^	[25]	3.24E-05	0.0401	0.0021	0.4452	0.3526
Chu et al.	15	[26]	0.0349	0.0094	0.2735	0.3624	0.3357
Kratz et al.	10^c^	[24]	9.18E-04	0.0201	0.0049	0.1277	0.0294
Chen et al.	94^d^	[27]	3.15E-04	0.7403	0.0008	0.0268	0.3455
Wan et al.	9^e^	[28]	0.0348	0.0643	0.0540	0.0411	0.0643

^a^Only adenocarcinoma patients were analyzed here.

^b^CPEB4 was missing in the probeset of Affymetrix U133A platform.

^c^WNT3A was missing in the probeset of Affymetrix U133A platform.

^d^Only 94 genes remained after removing redundant probes.

^e^MTX1 was missing in the probeset of Affymetrix U133A platform.

### Exploration of association between the 16-gene signature and drug response

Finally, we explored the ability of the 16-gene signature to serve as a predictive biomarker for drug response. To address this issue, we considered the microarrays of GSE36133 [15], which contained gene expression profiles and efficacy data of 23 drugs for 89 human lung cancer cell lines. The drug Irinotecan was not included in the analysis because the data were missing in more than 50% of the 89 cell lines. The expression data were retrieved from the GEO website and normalized by the quantile algorithm. Summarized pathway scores of identified genes were obtained by following the same procedures described previously. The efficacies of drugs on each cell line were represented by the variable activity area, with a higher value of the activity area representing higher inhibition ability of the corresponding drug in the cell line. The authors determined the activity area of a drug by measuring its proportion of relative growth inhibition compared to the untreated samples at different drug concentrations [15]. For each drug, a linear regression model was utilized to evaluate the association between the value of the activity area and the derived risk score. The results, summarized in Additional file 1: Table S6, indicated that the risk score was associated with three drugs, Nutlin-3, RAF215 and ZD-6474. Subsequently, the 89 cell lines were divided into three groups based on their scores and two non-parameter tests, Kruskal-Wallis test and Wilcoxon rank sum test, were performed. Only the drug, ZD-6474, targeting *EGFR*, showed significant differences in both statistical tests (Additional file 1: Figure S2), and better drug response was observed in the patients classified in the “High” group. This suggested that high-risk lung adenocarcinoma patients with poor survival may benefit from receiving ZD-6474 treatment. In summary, these results implied that our proposed pathway score is not only a prognostic biomarker for survival outcomes but also a predictive biomarker for selecting potentially useful drugs to treat lung adenocarcinoma patients.

## Discussion

**Figure 2:**
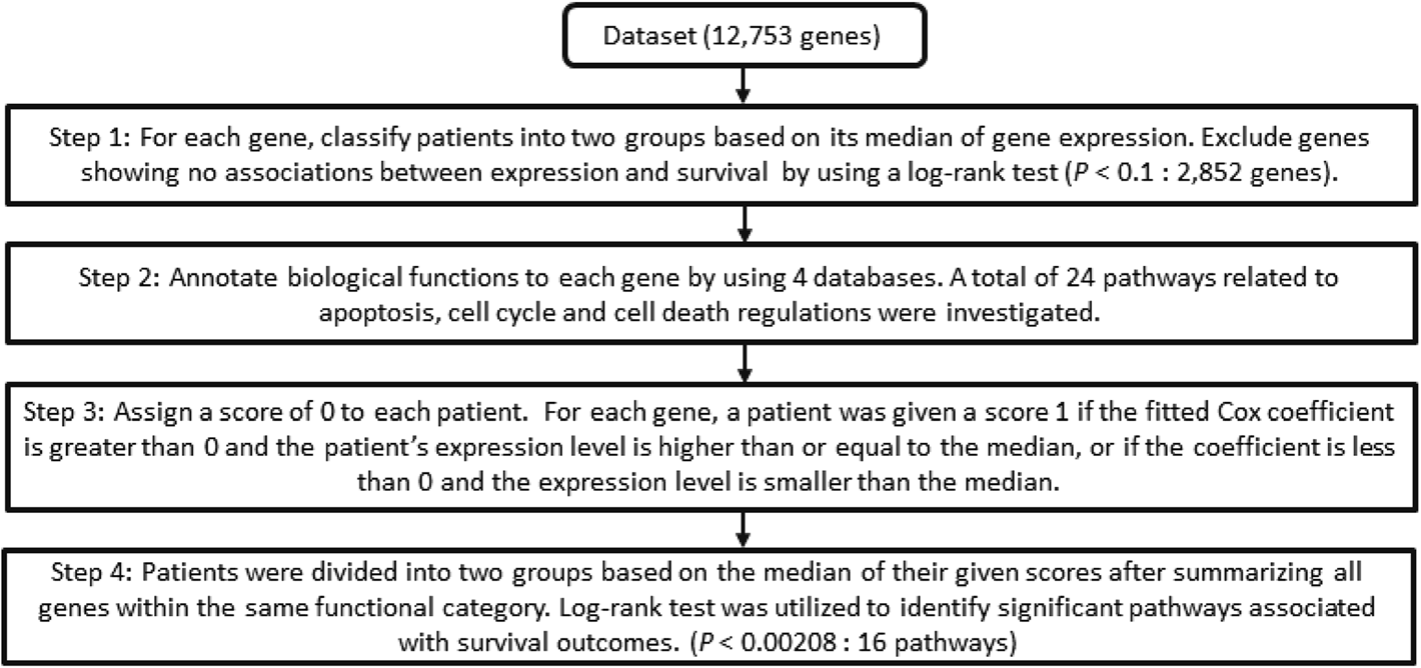
Flowchart for identifying predictive genes associated with survival outcomes in lung adenocarcinoma.

In the development of the 16-gene signature, the gene filtering procedure used the log-rank test to exclude genes showing no associations with survival outcomes (Step 1 in Figure 2). The p-value cut off was set at 0.1 in order to prevent excessive removal of genes. A similar strategy was utilized in a published report on ovarian cancer [8], in which the authors used a slightly looser threshold (*P* < 0.15) to retain the top quartile of associated genes. In our data, about 22% of the genes (2,852 out of 12,753) passed the threshold (*P* < 0.1). In other words, approximately 80% of the genes were removed by this step, which excluded the majority of genes examined by microarray. A more stringent cut off of *p*-values may help decrease the false-positive rates. However, two important questions may arise by using this approach. First, the proposed pathway-based analyses required a sufficient number of input genes in order to preserve the biological meaning of functional-relevant gene sets. Next, it is well-known that a huge discrepancy was observed across independent datasets, which may be attributed to different experimental protocols, distinct ethnicity, and dissimilar clinical parameters of investigated patients. Although a stringent threshold may provide better results for a single dataset, it often simultaneously increases the chance of overfitting the training model. Since the goal of this study is to identify predictive and reproducible genes across different studies, we prefer a slightly looser *p*-value cut off to retain information and the generality of results.

Four biological databases were utilized to provide functional annotations for the selected genes, and our results revealed that many cancer-related pathways were possible predictors for survival outcomes in lung adenocarcinoma (Table 1). Among them, an internal validation test excluded 5 pathways that showed low predictability. Furthermore, in the external validation, the pathway that was shown to be reproducible across 5 datasets does not have the smallest p-value from the log-rank test results. This implied that filtering survival predictors based purely on the p-value may not be the best strategy because such approach may cause overfitting of the model to the training dataset. In addition, lung cancer is such a heterogeneous disease and independent datasets had different ethnic characteristics, various experimental platforms, and distinct clinical features of patients. A pathway having the highest reproducibility across independent studies may be more applicable to clinic use than a pathway with the most significant p-value in a single dataset. Furthermore, for the 11 pathways shown in Table 2, we collected the most significant gene from each pathway into a gene set. The same scoring and evaluation procedures described previously were performed in 5 lung adenocarcinoma datasets by using this gene set. The result showed that its prediction performance and consistency were inferior to that obtained from the 16 genes, suggesting that using the union of the most significant gene across different pathways may be not the best choice.

Several previous studies have reported that the 16 genes identified in this study were highly related to the development, progression and treatment of cancer cells [20,29-38]. *DNM1L* was associated with poor survival in lung adenocarcinoma patients when co-expressing with *PKP3*[20], and nuclear expression of *DNM1L* was correlated with not only poor prognosis for lung adenocarcinoma but also drug resistance during hypoxia [29]. Cleavage of *DSG2* was mediated by junction opener 1 (JO-1), and downstream signaling combined with a monoclonal antibody targeting *EGFR*, Erbitux, provide better therapeutic outcomes in *EGFR*-positive lung cancer [30]. *DSP* has been reported as an overexpressing gene in high-risk patients with stage one non-small cell lung cancer (NSCLC), and is included in the 64-gene signature to predict survival outcomes [31]. The high mobility group 2 protein (*HMG2*) was able to increase the drug sensitivity of cisplatin in lung cancer cells by inhibiting repair of the DNA damage resulting from cisplatin [32]. A previous study demonstrated that *KPNB1* was overexpressed in cervical cancer cells, and decreased expression of *KPNB1* greatly reduced cell proliferation and triggered cell death procedures [33]. The expression of *LMNB1* was significantly up-regulated in the plasma and tissue of hepatocellular carcinoma patients, and it was associated with tumor stages, tumor sizes, and number of nodules [34]. Low mRNA and protein expression of *MAPT* was correlated with high expression of *TUBB3*, which showed prognostic significance with disease-free survival and overall survival in patients with early breast cancer [35]. *CLDN1* was a transcriptional activator of *OCLN*, and was also reported as not only a metastasis suppressor but also a possible prognostic predictor for lung adenocarcinoma [36]. *PAK2* was a necessary interaction partner of *ARHGDIB*, and knockdown of *PAK2* greatly reduced *ARHGDIB*-induced cell invasion and *ARHGDIB*-mediated chemoresistance in gastric cancer [37], and loss of *SATB1* in lung cancer has been shown as a possible marker for poor survival [38].

Identification of possible prognostic biomarkers for various cancers has been widely investigated in the past two decades, and several successful studies have been reported [8,24,39,40]. For instance, the MammaPrint® Test divided breast cancer patients into high and low risk groups for distant metastases within 10 years by examining the expression signature of 70 genes [39,40], and it has been the only signature approved by the FDA for clinical use. Regarding lung cancer, in addition to the irreproducibility of identified biomarkers across independent datasets [7], gene expression profiles obtained from different microarray platforms pose another challenge. A predicting model developed using one microarray platform usually cannot be directly utilized in another microarray platform due to differences in their dynamic ranges of intensity values and lack of a normalization algorithm to adjust them to the same baseline. Therefore, a predictive model will become more useful when it is not necessary to rebuild the model using different parameters in independent datasets. In this study, the medians of proposed scores in the 5 lung adenocarcinoma datasets were 8, except for the Beer dataset. The median score in the Beer dataset was 7 based on only 14 genes studied, since two genes, *DSP* and *STK24*, were not examined in the corresponding microarray platform. In summary, our approach provides a consistent cutting threshold across different microarray platforms, and thus may enable application to independent studies.

To further investigate the proposed analysis procedures in this study, Cox hazard regression models were used to evaluate the top 5 pathways shown in Table 2. Generally, the risk scores obtained from the other 4 pathways were significantly associated with survival outcomes in at least 2 datasets (*P* < 0.05), suggesting that our approach is effective in selection of predictive genes from the whole gene pool. In addition, we followed the same procedures described previously and performed the three statistical tests to evaluate the drugs associated with the scores obtained from the second pathway, the Biocarta cell cycle pathway. The scores were significantly correlated with three drugs using a linear regression model (*P* < 0.05), and two of them, Erlotinib and Lapatinib, also passed the Kruskal-Wallis and Wilcoxon rank sum tests. Unexpectedly, these two drugs both target *EGFR*, concurring that treating lung cancer patients using *EGFR* inhibitors is a feasible approach. Therefore, our proposed methodology may facilitate classification of patients into different groups based on their drug responses.

Identification of prognostic biomarkers for lung cancer is useful but it may become more valuable if the predicting signature can simultaneously reveal the drug response. Although ZD-6474, also known as Vandetanib, targeting *EGFR*, has not been approved for treatment of NSCLC, it has been used in medullary thyroid cancer [41]. One randomized phase 2 clinical trial demonstrated the efficacy of Vandetanib in locally advanced or metastatic differentiated thyroid cancer [42]; another meta-analysis study including 14 trials indicated that NSCLC patients treated by Vandetanib showed better progression-free survival, especially the adenocarcinoma subtype [43]. This suggests that Vandetanib has high potential for approval and use in treatment of patients with lung cancer [41]. Therefore, since the efficacy of Vandetanib was associated with the score proposed to summarize the 16 genes (Additional file 1: Figure S2), this signature may serve as both prognostic and predictive biomarkers for lung adenocarcinoma patients in the future.

To further explore the potential of the 16-gene signature used as a predictive biomarker, we evaluated its performance by analyzing another NSCLC dataset with treatment information, GSE14814 [26]. The series matrix file of GSE14814 was retrieved from the GEO database. There were 90 lung cancer patients in the series matrix file but only 28 adenocarcinoma patients. We applied the same procedure to derive the risk scores of each lung adenocarcinoma patient. Unfortunately, no significant difference between “High” and “Low” groups was observed in the 28 lung adenocarcinoma patients using the 16-gene signature (*P* > 0.05). Among the 28 lung adenocarcinoma patients, there are 17 treated patients and 11 controls. Again, no significant differences were observed in these two sub-groups. These insignificant results might be attributed to the small sample size (N = 28). The 17 treated patients were insufficient for subgroup analysis of difference between high and low risk groups. In addition, we evaluated the performance of the 16-genes on the 52 lung squamous cell carcinoma patients. The result was similar to the previous analysis that the 16-gene signature can’t significantly predict the squamous cell carcinoma patients. The 16-gene signature cannot serve as a predictive signature in GSE14814.

## Conclusions

In this study, we identified 16 predictive genes for lung adenocarcinoma patients via pathways. Four widely used biological databases were utilized to provide the functional annotations of genes. The risk scores derived from these 16 genes were reproduced in 4 independent studies, which account for a total of more than 700 lung adenocarcinoma patients. However, the risk scores showed no predictive ability for lung squamous cell carcinoma, suggesting that these 16 genes were subtype-specific for lung cancer. Lastly, statistical tests showed that the 16-gene signature was associated with the efficacy of an anti-cancer drug, ZD-6474, directly targeting *EGFR*. Therefore, these 16 genes may serve as not only prognostic biomarkers but also predictive biomarkers for lung adenocarcinoma, and have potential for clinical use in the future.

## Methods

### Processing of the training set

The microarray dataset from the study of Shedden et al. [5] was utilized as the training set to develop a prognostic signature for survival risk prediction. This dataset provided not only the largest number of lung adenocarcinoma patients currently in the world, but also comprehensive clinical information for further comparisons. The raw cel files of Affymetrix microarray retrieved from the website were imported into the Partek Genomics Suite version 6.4 to perform the pre-processing steps, including robust multiarray average and the quantile normalization method. After pre-processing, the gene symbol of each probe was obtained by searching the official annotation file version 32 provided by Affymetrix, Inc. Only probes annotated with one single gene remained for further analyses and the expression values of multiple probes showing the same gene symbol were summarized by a single value by using their mean. Consequently, a total of 12,753 genes were investigated in this study.

### Identification of potential gene signatures by biological pathways

The procedure to identify significant survival predictors is illustrated in Figure 2. Specifically, for each gene, patients were divided into two groups based on the median of expression values. A patient was classified into the “High” group if his/her expression level was greater than or equal to the median and into the “Low” group if his/her expression level was less than the median. Next, the log-rank test was performed to compare the “High” versus “Low” groups for each gene. The significant genes were selected based on a pre-determined statistical criterion at p < 0.10 (Step 1).

Four biological databases, including KEGG [44], BioCarta, Pathway Interaction Database (PID) [45] and Reactome [46], were utilized to provide functional annotations of selected genes. Because it is well-known that apoptosis, cell cycle and cell death regulations are dominant players in regulating cancer cells [47,48], we selected 24 pathways that were related to these three functions for further investigations (Step 2).

The associations between gene expression and survival were evaluated using the Cox hazard regression model for each gene selected from Step 2 (24 pathways). Initially, a score of 0 was assigned to each patient. For each gene, a score of 1 was given to a patient 1) if the fitted Cox coefficient was greater than 0 and the patient’s expression level was greater or equal to the median, or 2) the Cox coefficient was less than 0 and the expression level was less than the median. The total risk score of each patient was computed by summing all genes within the same pathway (Step 3).

For each pathway, patients were classified into two groups based on the median of the risk scores. The log-rank test for difference between the two groups was performed; the pathway-specific significant signature sets were identified at α = 0.05/24 (Step 4). The significant signature sets were analytically evaluated to assess their predictability and generalizability.

### Availability of supporting data

Shedden’s dataset: https://array.nci.nih.gov/caarray/project/details.action?project.id=182.

## Abbreviations

GEO: Gene expression omnibus; NSCLC: Non-small cell lung cancer; PID: Pathway interaction database.

## Competing interests

The authors declare that they have no competing interests.

## Authors’ contributions

TPL, EYC and JJC conceived the study. TPL and JJC developed the methodology, performed the analysis and wrote the manuscript. All authors read and approved the final manuscript.

## Supplementary Material

Additional file 1: Table S1 Identified significant gene members (*P* < 0.1) showing associations to the survival outcomes in the 24 pathways investigated. **Table S2**: Characteristics of the six microarray datasets. **Table S3**: Prediction performances of the 11 pathways in lung squamous cell carcinoma. **Table S4**: Characteristics of the 442 lung adenocarcinoma patients analyzed in the study of Shedden et al. **Table S5**: Cox hazard regression model of scores and clinical variables in the study of Shedden et ala. **Table S6**: Linear regression analysis of the activity area of 23 drugs on the risk scores derived from the 16-gene signature. **Figure S1**: Kaplan-Meier survival curves of lung adenocarcinoma patients classified into three groups. For each dataset, the patients were classified into “High”, “Intermediate” and “Low” groups according to their summarized scores. **(a)** Shedden et al. **(b)** GSE3141 **(c)** GSE8894 **(d)** GSE11969 **(e)** Beer et al. **Figure S2**: Lung cancer cell lines with higher scores associated with higher activity area of ZD-6474. The 89 lung cancer cell lines from GSE36133 were divided into “High”, “Intermediate” and “Low” groups based on their scores. Box plot was utilized to illustrate their activity areas among three groups. Kruskal-Wallis test and Wilcoxon rank sum test were performed to assess their statistical differences. Click here for file
