# Supplementary material for: Identification of reproducible gene expression signatures in lung adenocarcinoma
Source: BMC Bioinformatics. 2013 Dec 26;14:371. doi: 10.1186/1471-2105-14-371 (PMC3877965; doi:10.1186/1471-2105-14-371)

Supplementary data

Table S1. Identified significant gene members ($P < 0.1$) showing associations to the survival outcomes in the 24 pathways investigated.

Name	Database	Number of significant genes	Symbol
**BIOCARTA CELLCYCLE PATHWAY**	BioCarta	7	CCNB1|CCND3|CCNE1|CDK2|CDKN1A|RBL1|TFDP1|
**BIOCARTA DEATH PATHWAY**	BioCarta	9	APAF1|BCL2|BID|CHUK|CYCS|MAP3K14|TNFRSF25|TNFSF12|TRADD|
**BIOCARTA TCAPOPTOSIS PATHWAY**	BioCarta	2	CD3E|CD3G|
**Apoptosis KEGG**	KEGG	16	APAF1|BAD|BCL2|BID|CHUK|CYCS|IKBKG|IRAK1|MAP3K14|PIK3CD|PIK3CG|PIK3R1|PRKACA|PRKACG|PRKAR1B|TRADD|
**Cell cycle**	KEGG	46	ANAPC1|ANAPC10|ANAPC5|BUB1B|BUB3|CCNA2|CCNB1|CCNB2|CCND3|CCNE1|CCNE2|CDC14A|CDC20|CDC25C|CDC27|CDC6|CDC7|CDK2|CDKN1A|CDKN1C|CHEK1|CREBBP|CUL1|DBF4|E2F4|ESPL1|GADD45G|MAD2L1|MCM2|MCM3|MCM4|MCM6|MCM7|PCNA|PLK1|PRKDC|PTTG1|RAD21|RBL1|TFDP1|TGFB1|TGFB3|TTK|YWHAH|YWHAQ|YWHAZ|
**Caspase cascade in apoptosis**	PID	9	APAF1|ARHGDIB|BCL2|BID|CYCS|DIABLO|MADD|SATB1|TRADD|
**Polo-like kinase signaling events in the cell cycle**	PID	42	ARHGEF7|ARRB2|AURKA|BIRC5|BRCA1|BUB1B|CCNB1|CCNE1|CDC20|CDC25C|CENPA|CENPE|CHUK|DLGAP5|ECT2|ERC1|ERCC6L|GORASP1|IKBKG|KIF20A|MAP3K14|MLF1IP|NDC80|NDEL1|NFKB2|NUDC|PAK1|PIK3R1|PLK1|PLK2|PPP1CB|PPP2R1A|PPP2R5D|PRKACA|PRKCA|RAB1A|RAN|RELB|TACC3|TPX2|TUBG1|XPO1|
**APC C-mediated degradation of cell cycle proteins**	Reactome	32	ANAPC1|ANAPC10|ANAPC5|AURKA|BUB1B|BUB3|CCNA2|CCNB1|CDC14A|CDC20|CDC27|CDK2|CUL1|MAD2L1|PLK1|PSMA3|PSMA4|PSMA5|PSMA6|PSMA7|PSMB5|PSMC5|PSMC6|PSMD1|PSMD12|PSMD14|PSMD2|PSMD9|PTTG1|UBB|UBE2C|UBE2D1|
**Apoptosis induced DNA fragmentation**	Reactome	2	HMGB2|KPNB1|
**Apoptosis Reactome**	Reactome	43	ADD1|APAF1|APPL1|ARHGAP10|BAD|BCL2|BID|CYCS|DAPK2|DIABLO|DNM1L|DSG2|DSP|DYNLL1|HMGB2|KPNB1|LMNB1|MAPT|OCLN|PAK2|PKP1|PMAIP1|PRKCD|PRKCQ|PSMA3|PSMA4|PSMA5|PSMA6|PSMA7|PSMB5|PSMC5|PSMC6|PSMD1|PSMD12|PSMD14|PSMD2|PSMD9|SATB1|STK24|TFDP1|TJP1|TRADD|UBB|
**Apoptotic cleavage of cell adhesion proteins**	Reactome	5	DSG2|DSP|OCLN|PKP1|TJP1|
**Apoptotic cleavage of cellular proteins**	Reactome	12	ADD1|DSG2|DSP|LMNB1|MAPT|OCLN|PKP1|PRKCD|PRKCQ|SATB1|STK24|TJP1|
**Apoptotic execution**	Reactome	16	ADD1|DNM1L|DSG2|DSP|HMGB2|KPNB1|LMNB1|MAPT|OCLN|PAK2|PKP1|PR

**phase**			KCD|PRKCQ|SATB1|STK24|TJP1|
**Apoptotic factor-mediated response**	Reactome	3	APAF1|CYCS|DIABLO|
**Cell Cycle Mitotic**	Reactome	60	AHCTF1|ANAPC1|ANAPC10|ANAPC5|APITD1|AURKA|BIRC5|BUB1B|BUB3|CCDC99|CCNA2|CCNB1|CCNB2|CCND3|CCNE1|CCNE2|CDC14A|CDC20|CDC25C|CDC27|CDC6|CDC7|CDCA8|CDK11A|CDK2|CDKN1A|CENPA|CENPE|CENPF|CENPI|CENPN|CENPQ|CEP152|CEP164|CEP192|CKS1B|CUL1|DBF4|DHFR|DNA2|DSN1|DYNC1I2|DYNLL1|E2F4|ERCC6L|FEN1|GINS1|GINS2|GINS4|GMNN|GORASP1|KIF20A|KIF23|KIF2C|KNTC1|MAD2L1|MAPRE1|MCM10|MCM2|MCM3|
**Cell death signalling via NRAGE NRIF and NADE**	Reactome	8	AKAP13|ARHGEF17|ARHGEF2|ARHGEF7|BAD|ECT2|NET1|UBB|
**Death Receptor Signalling**	Reactome	1	TRADD|
**Extrinsic Pathway for Apoptosis**	Reactome	1	TRADD|
**Intrinsic Pathway for Apoptosis**	Reactome	9	APAF1|BAD|BCL2|BID|CYCS|DIABLO|DYNLL1|PMAIP1|TFDP1|
**NRAGE signals death through JNK**	Reactome	7	AKAP13|ARHGEF17|ARHGEF2|ARHGEF7|BAD|ECT2|NET1|
**NRIF signals cell death from the nucleus**	Reactome	1	UBB|
**Regulation of Apoptosis**	Reactome	18	APPL1|ARHGAP10|DAPK2|PAK2|PSMA3|PSMA4|PSMA5|PSMA6|PSMA7|PSMB5|PSMC5|PSMC6|PSMD1|PSMD12|PSMD14|PSMD2|PSMD9|UBB|
**Regulation of mitotic cell cycle**	Reactome	32	ANAPC1|ANAPC10|ANAPC5|AURKA|BUB1B|BUB3|CCNA2|CCNB1|CDC14A|CDC20|CDC27|CDK2|CUL1|MAD2L1|PLK1|PSMA3|PSMA4|PSMA5|PSMA6|PSMA7|PSMB5|PSMC5|PSMC6|PSMD1|PSMD12|PSMD14|PSMD2|PSMD9|PTTG1|UBB|UBE2C|UBE2D1|
**Role of DCC in regulating apoptosis**	Reactome	2	APPL1|DAPK2|

Table S2. Characteristics of the six microarray datasets.

Dataset	Platform	# of AD	# of SQ	# of Total	Ref
Shedden et al.	Affymetrix U133A	442	0	442	(5)
Beer et al.	Affymetrix HuGeneFL	86	0	86	(3)
GSE3141	Affymetrix U133plus2.0	58	53	111	(4)
GSE4573	Affymetrix U133plus2.0	0	130	130	(26)
GSE8894	Affymetrix U133plus2.0	63	75	138	(25)
GSE11969	Agilent Homo sapiens 21.6K	90	35	125	(27)
Total	---	738	293	1,031	---

AD : adenocarcinoma, SQ : Squamous

Table S3. Prediction performances of the 11 pathways in lung squamous cell carcinoma.

Pathway (Gene Number)	Database	Squamous Cell Carcinoma			
		GSE3141	GSE4573	GSE8894	GSE11969
Apoptotic execution phase (16)	Reactome	0.4173	0.3441	0.5646	0.5338
BIOCARTA CELLCYCLE PATHWAY (7)	Biocarta	0.5678	0.3063	0.6809	0.3636
Cell cycle (46)	KEGG	0.6601	0.6102	0.0759	0.7677
Apoptosis (16)	KEGG	0.8923	0.3092	0.1139	0.7981
Caspase cascade in apoptosis (9)	PID	0.502	0.6175	0.1220	0.9971
APC C-mediated degradation of cell cycle proteins (32)	Reactome	0.8796	0.8088	0.0911	0.8097
Regulation of mitotic cell cycle (32)	Reactome	0.8796	0.8088	0.0911	0.8097
Polo-like kinase signaling events in the cell cycle (42)	PID	0.9086	0.8422	0.0584	0.655
Intrinsic Pathway for Apoptosis (9)	Reactome	0.9537	0.4451	0.0581	0.3487
Apoptosis (43)	Reactome	0.7707	0.8566	0.1138	0.6978
Cell Cycle Mitotic (60)	Reactome	0.3409	0.4937	0.2609	0.9444

Table S4. Characteristics of the 442 lung adenocarcinoma patients analyzed in the study of Shedden et al.

Variables	Sample (%)	Variable definition in Cox hazard regression model
**Age**		
**≥65**	226 (51.13%)	1
**<65**	216 (48.87%)	0
**Gender**		
**Male**	228 (51.58%)	1
**Female**	214 (48.42%)	0
**Smoking**		
**Smoker**	301 (68.10%)	1
**Never Smoker**	48 (10.86%)	0
**NA**	93 (21.04%)	NA
**Grade**		
**Well**	53 (11.99%)	0
**Moderate**	203 (45.93%)	1
**Poor**	175 (39.59%)	2
**NA**	11 (2.49%)	NA

Table S5. Cox hazard regression model of scores and clinical variables in the study of Shedden et al[a].

Variable	β	Exp(β)	95% confidence interval	*p*-value
Age	0.388	1.473	1.089-1.994	1.20E-02
Gender	0.07	1.072	0.787-1.461	6.58E-01
Smoking	0.117	1.123	0.691-1.828	6.38E-01
Grade	0.036	1.036	0.810-1.327	7.74E-01
Score	0.108	1.114	1.062-1.168	8.16E-06

[a] A total of 102 patients were excluded due to their missing data in clinical variables

Table S6. Linear regression analysis of the activity area of 23 drugs on the risk scores derived from the 16-gene signature.

Drug	Target	Coefficient	p-value[a]	Drug	Target	Coefficient	p-value[a]
17-AAG	*HSP90*	-0.045	0.362	PD-0325901	*MEK*	-0.05	0.347
AEW541	*IGF1R*	-0.032	0.143	PD-0332991	*CDK4*	-0.032	0.173
AZD0530	*ABL*	0.043	0.196	PF2341066	*c-MET*	-0.045	0.11
AZD6244	*MEK*	-0.065	0.102	PHA-665752	*c-MET*	0.009	0.703
Erlotinib	*EGFR*	0.033	0.402	PLX4720	*RAF*	0.006	0.701
L-685458	*GS*	0.006	0.676	RAF265	*RAF*	-0.07	0.03
Lapatinib	*EGFR*	-0.02	0.501	Sorafenib	*RTK*	0.001	0.938
LBW242	*XIAP*	-0.025	0.441	TAE684	*ALK*	-0.018	0.598
Nilotinib	*ABL*	-0.012	0.545	TKI258	*FGFR*	-0.038	0.066
Nutlin-3	*MDM2*	-0.042	0.021	Topotecan	*TOP2*	-0.081	0.143
Paclitaxel	*TUBB1*	-0.069	0.247	ZD-6474	*EGFR*	0.069	0.035
Panobinostat	*HDAC*	-0.032	0.305				

[a] Estimated by using wilcoxon rank sum test

Figure S1. Kaplan-Meier survival curves of lung adenocarcinoma patients classified into three groups. For each dataset, the patients were classified into “High”, “Intermediate” and “Low” groups according to their summarized scores. (a) Shedden et al. (b) GSE3141 (c) GSE8894 (d) GSE11969 (e) Beer et al.

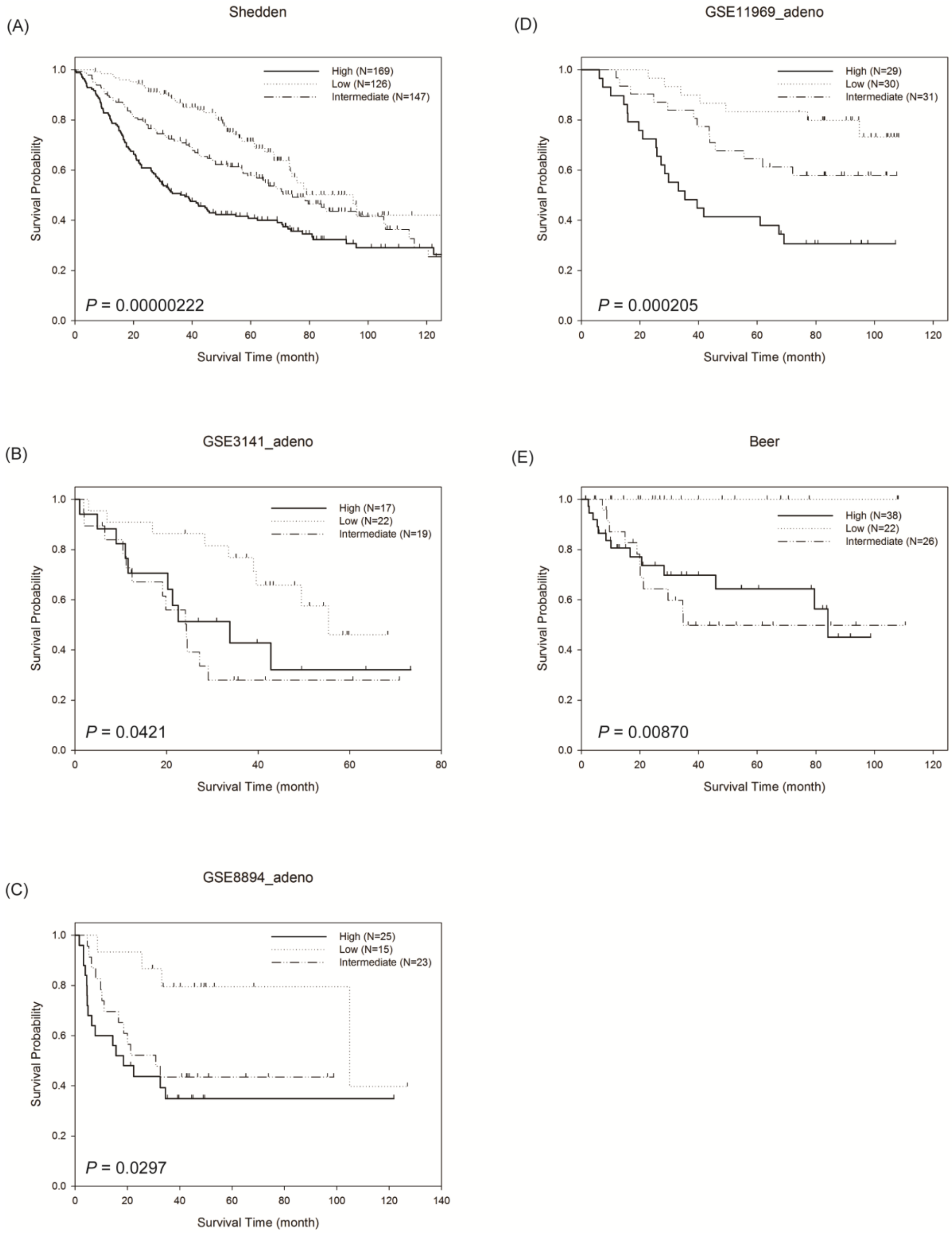

Figure S2. Lung cancer cell lines with higher scores associated with higher activity area of ZD-6474. The 89 lung cancer cell lines from GSE36133 were divided into “High”, “Intermediate” and “Low” groups based on their scores. Box plot was utilized to illustrate their activity areas among three groups. Kruskal-Wallis test and Wilcoxon rank sum test were performed to assess their statistical differences. (*: P <0.05)

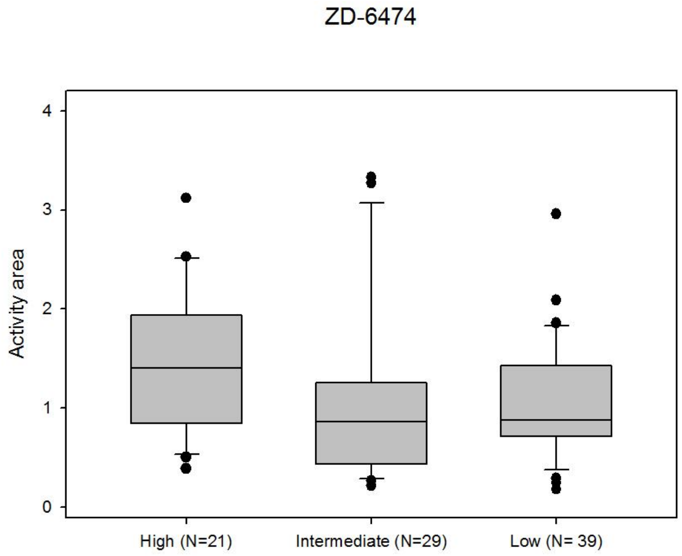

Supplement: Additional file 1: Table S1 — Identified significant gene members (P < 0.1) showing associations to the survival outcomes in the 24 pathways investigated. Table S2: Characteristics of the six microarray datasets. Table S3: Prediction performances of the 11 pathways in lung squamous cell carcinoma. Table S4: Characteristics of the 442 lung adenocarcinoma patients analyzed in the study of Shedden et al. Table S5: Cox hazard regression model of scores and clinical variables in the study of Shedden et ala. Table S6: Linear regression analysis of the activity area of 23 drugs on the risk scores derived from the 16-gene signature. Figure S1: Kaplan-Meier survival curves of lung adenocarcinoma patients classified into three groups. For each dataset, the patients were classified into “High”, “Intermediate” and “Low” groups according to their summarized scores. (a) Shedden et al. (b) GSE3141 (c) GSE8894 (d) GSE11969 (e) Beer et al. Figure S2: Lung cancer cell lines with higher scores associated with higher activity area of ZD-6474. The 89 lung cancer cell lines from GSE36133 were divided into “High”, “Intermediate” and “Low” groups based on their scores. Box plot was utilized to illustrate their activity areas among three groups. Kruskal-Wallis test and Wilcoxon rank sum test were performed to assess their statistical differences. [file 1471-2105-14-371-S1.pdf]
